# Supplementary material for: Genetics of growth rate in induced pluripotent stem cells
Source: bioRxiv. 2025 Jul 3:2025.07.02.662844. Preprint. [Version 1] doi: 10.1101/2025.07.02.662844 (PMC12236597; doi:10.1101/2025.07.02.662844)
Supplement: 1 [file NIHPP2025.07.02.662844V1-supplement-1.pdf]

bioRxiv preprint doi: https://doi.org/10.1101/2025.07.02.662844; this version posted July 3, 2025. The copyright holder for this preprint (which was not certified by peer review) is the author/funder. This article is a US Government work. It is not subject to copyright under 17 USC 105 and is also made available for use under a CC0 license.

Supplemental Tables

ESM Table 1. Summary statistics of 602 iPSC line attributes.

**Sex**	
Male	278
Female	324
**Media**	
mTeSR	347
Freedom	255
**Starting cell type**	
Blood	149
Fibroblast	453
**Reprogramming method**	
mRNA	240
Sendai virus	362
**Ancestry group**	
1KG-EUR-like	446
1KG-EAS-like	51
1KG-AMR-like	44
1KG-AFR-like	39
1KG-SAS-like	22

bioRxiv preprint doi: https://doi.org/10.1101/2025.07.02.662844; this version posted July 3, 2025. The copyright holder for this preprint (which was not certified by peer review) is the author/funder. This article is a US Government work. It is not subject to copyright under 17 USC 105 and is also made available for use under a CC0 license.

ESM Table 2. *WDR54* variants from the rare variant association study. Genomic coordinates from GRCh38. Source of genotype DNA reported in columns: ”Blood DNA”, ”Fibroblast DNA”, and ”iPSC DNA.”

rsID	Variant Location	Variant Type	CADD	Blood DNA	Fibroblast DNA	iPSC DNA
rs147527356	chr2:74422334	Missense	22.5	0	0	2
rs150243972	chr2:74424959	Missense	21.8	0	2	0
rs187456631	chr2:74425080	Missense	25.2	0	4	0
rs139454931	chr2:74425113	Missense	22.2	0	0	4
rs140810540	chr2:74425208	Missense	25.9	0	0	5

bioRxiv preprint doi: https://doi.org/10.1101/2025.07.02.662844; this version posted July 3, 2025. The copyright holder for this preprint (which was not certified by peer review) is the author/funder. This article is a US Government work. It is not subject to copyright under 17 USC 105 and is also made available for use under a CC0 license.

Supplemental Figures

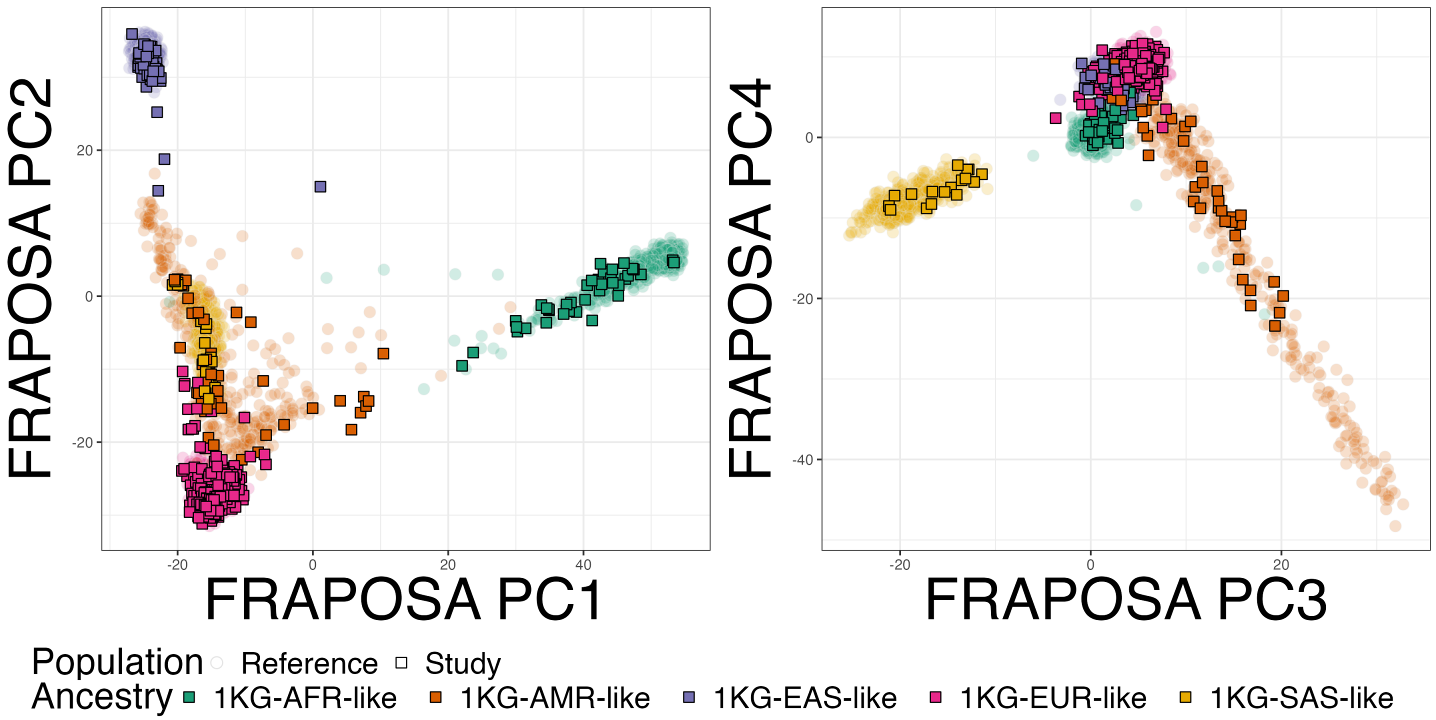

ESM Fig. 1. Genetic diversity represented in cohort. First four genetic PCs and predicted genetic ancestry group from FRAPOSA analysis (Methods). Color indicates genetic ancestry group (1KG-AFR-like, 1KG-AMR-like, 1KG-EAS-like, 1KG-EUR-like, or 1KG-SAS-like). Left-hand side plot displays principal components 1 versus 2; right-hand side plot displays principal components 3 versus 4. Point shape indicates study samples (squares) and reference panel samples (circles).
